# Medical Journals

**Published:** 1841-10

**Authors:** 


### MEDICAL JOURNALS.

We sometimes meet with the complaint in the papers of our contemporaries, that the Medical journals of the United States are too numerous. It may be that there are more than are well supported, either by the pens or the purses of the profession, but we are sure there are not more than the profession ought to support. What physycian can hope to keep pace with the progress of medical science, who is not the reader of at least one medical journal? As well might the politician attempt to dispense with political newspapers. And yet, in every quarter, we meet with practitioners who are not only not subscribers to any journal of medicine, but who are not in the way of ever being one. Without persevering study, it is admitted, no one can become eminent in the medical profession, and nothing is a greater provocation to reading than these periodicals, which, coming to us monthly or quarterly, indicate the improvements and discoveries going forward in medicine in all parts of the world. Y.

